# RefSeq database growth influences the accuracy of *k*-mer-based lowest common ancestor species identification

**DOI:** 10.1186/s13059-018-1554-6

**Published:** 2018-10-30

**Authors:** Daniel J. Nasko, Sergey Koren, Adam M. Phillippy, Todd J. Treangen

**Affiliations:** 1 Center for Bioinformatics and Computational Biology, University of Maryland, College Park, MD USA; 2 Genome Informatics Section, Computational and Statistical Genomics Branch, National Human Genome Research Institute, Bethesda, MD USA; 3 Department of Computer Science, Rice University, Houston, TX USA

**Keywords:** Taxonomic classification, Reference database, Metagenomics, Microbiome, Comparative analysis, *k*-mer, LCA

## Abstract

In order to determine the role of the database in taxonomic sequence classification, we examine the influence of the database over time on *k*-mer-based lowest common ancestor taxonomic classification. We present three major findings: the number of new species added to the NCBI RefSeq database greatly outpaces the number of new genera; as a result, more reads are classified with newer database versions, but fewer are classified at the species level; and Bayesian-based re-estimation mitigates this effect but struggles with novel genomes. These results suggest a need for new classification approaches specially adapted for large databases.

## Introduction

Fundamental questions of a metagenomic survey are (i) what microbes are present in each sample, (ii) how abundant is each organism identified in a sample, (iii) what role might each microbe play (i.e., what gene functions are present), and (iv) how do the previous observations change across samples and time. Specifically, there have been numerous studies highlighting the utility of metagenomic datasets for pathogen detection, disease indicators, and health [1, 2]. Addressing each of these fundamental questions is predicated on the ability to assign taxonomy and gene function to unknown sequences.

Several new tools and approaches for taxonomic identification of DNA sequences have emerged [3–5], in addition to community-driven “bake-offs” and benchmarks [6]. *k*-mer-based classification methods such as Kraken or CLARK [3, 7] are notable for their exceptional speed and specificity, as both are capable of analyzing hundreds of millions of short reads (ca. 100–200 base pairs) in a CPU minute. These *k*-mer-based algorithms use heuristics to identify unique, informative, *k*-length subsequences (*k*-mers) within a database to help improve both speed and accuracy. A challenge for *k*-mer-based classification approaches is that closely related species and strains often contain many identical sequences within their genomes. This challenge is typically addressed by assigning the query sequence with the lowest common ancestor (LCA [8]) of all species that share the sequence. A comprehensive benchmarking survey indicated that Kraken offered the best *F*_1_ score (a measure considering both precision and recall) among the *k*-mer-based taxonomic classifiers evaluated at the species level [9]. Bracken, a Bayesian method that refines Kraken results, is capable of estimating how much of each species is present among a set of ambiguous species classifications by probabilistically re-distributing reads in a taxonomic tree [10]. We thus selected Kraken and Bracken as representative tools from the genre of *k*-mer-based classification methods. The focus of this study was not to examine a specific software tool, but rather to evaluate the performance of a representative *k*-mer-based method given database variability over time.

Available *k*-mer-based methods for taxonomic identification and microbiome profiling rely on existing reference databases. While several investigations have examined the influence of contamination in specific database releases and identified idiosyncrasies specific to a release [11, 12], no study has examined the specific influence of perhaps the most popular database from which to build classification databases, the repository of sequenced, and assembled microbes (RefSeq), across various releases of the database. Additionally, metagenomic classification and profiling tools are commonly compared to each other using simulated datasets on a fixed database, with leave-one-out analysis, but never compared to each other across recent trajectories in database growth. The aim of this study was to elucidate the influence of RefSeq database growth over time on the performance of taxonomic identification using *k*-mer-based tools.

We measured the growth of the bacterial fraction of the RefSeq database in terms of both size and diversity. As the database grew, we found that fewer species-level classifications were attained while the fraction of genus-level classifications increased. This is a consequence of the LCA approach, whereby a shared sequence is assigned to the lowest common ancestor among the set of matching taxa. Thus, while we only evaluated Kraken and Bracken in this study, the challenges of RefSeq database growth stretch beyond *k*-mer-based classification methods and are likely to affect other LCA-based approaches.

## Results

### RefSeq database growth and diversity

**Fig. 1:**
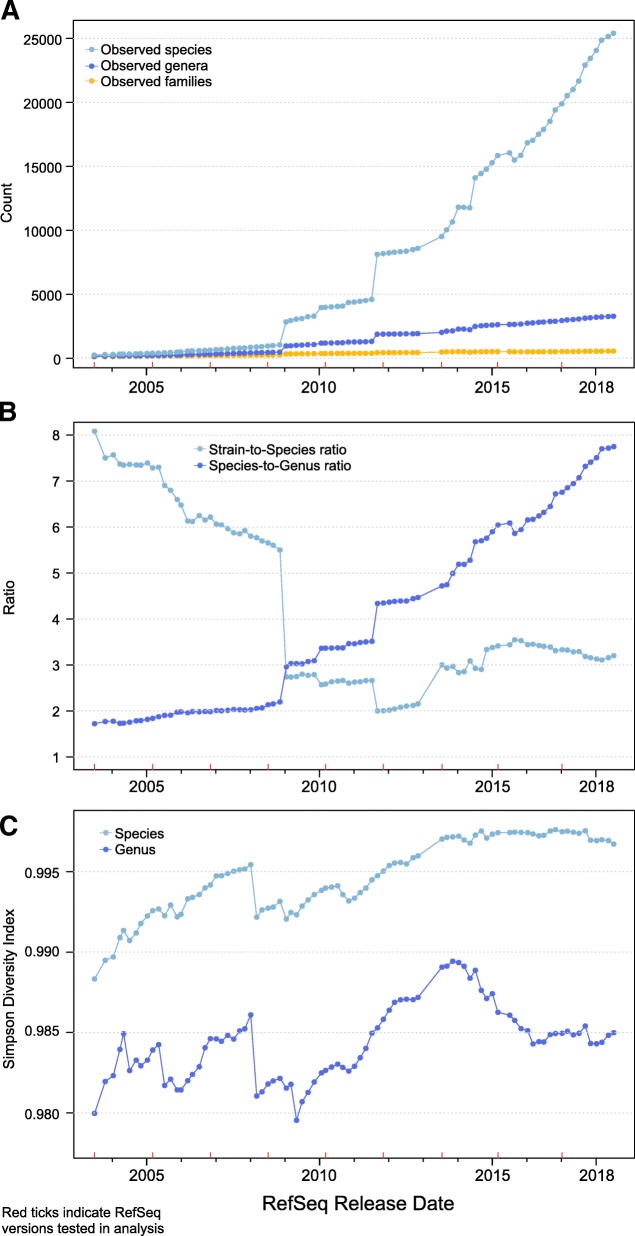
The diversity of genera has decreased in the majority of releases since November 2013. **a** The number of observed species has outpaced the number of observed genera, which has in turn outpaced the number of families as RefSeq has grown. **b** The ratio of strains-to-species has tended to decrease while the ratio of species-to-genera has tended to increase as RefSeq has grown. **c** Simpson’s diversity index of species in bacterial RefSeq has tended to increase every release (appearing to have plateaued recently), while Simpson’s diversity of genera tended to increase until November 2013, where it has tended to decrease

Since its first release in June 2003, bacterial RefSeq, on average, has doubled in size (giga base pairs, Gbp) every 1.5 years, with the number of unique 31-mers in the database growing at a similar rate. A more recent release, bacterial RefSeq version 89 (released 7/9/2018), totaled nearly 938 Gbp of sequence data. The number of observed species in RefSeq doubled nearly every 3 years (Fig. 1a), while the number of observed genera has not doubled in nearly 6 years (last doubling event was in September 2012). This gap in species and genus growth, albeit expected given the hierarchical nature of taxonomy, has led to a steady increase in the ratio of species-to-genera over time (Fig. 1b), growing from below two species to every one genus (version 1) to nearly eight species to every one genus (version 89). There is also a general, though fluctuating, decrease in the ratio of strains-to-species (Fig. 1b), declining from eight strains to one species (version 1) to approximately three strains to one species (version 89).

Simpson’s index of diversity is a metric with values between zero and one that reports the probability that two individuals randomly selected from a sample will not belong to the same taxonomic unit. Samples with high Simpson’s index of diversity (i.e., closer to one) may be considered more diverse than those with low values (i.e., closer to zero). The diversity for each version of the bacterial RefSeq was measured at the species and genus levels (Fig. 1c). The diversity of species tended to increase as the database grew (though it appears to have plateaued recently), while the diversity of genera peaked in November 2013, where it then declined and has not returned since. We suspect this is due to recent sequencing efforts that have focused on a handful of pathogenic species for outbreak detection [13].

Every release of the bacterial fraction of the RefSeq database resulted in more bases in the database. However, three releases resulted in fewer observed species and several resulted in decrease in species- and genus-level diversity (Fig. 1). Some of these shifts can be explained by the restructuring of RefSeq at certain releases. Versions 57–59 (Jan–Mar 2013) of RefSeq included drastic expansions of bacterial genomes as more microbial genomes that represent complete or draft assemblies from novel isolates and clinical and population samples were added during this period. Indeed, the addition of clinically relevant bacteria was substantial and led to the most abundant genera changing from *Bacillus* prior to the expansion to *Pseudomonas* and *Streptomyces* post-expansion. Release 65 (May 2014) saw the creation of the “archaea” and “bacteria” classifications, breaking apart what was once the “microbial” classification.

### Taxonomic classification over time with a simulated metagenome

**Fig. 2:**
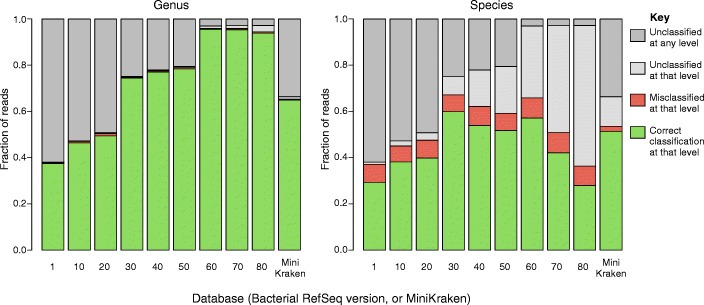
Fraction of correct species classifications (right) decreases in more recent RefSeq database versions and instead are classified at the genus level (left). Kraken classification results of simulated reads from known genomes against nine versions of the bacterial RefSeq database and the MiniKraken database. Misclassifications at the genus and species levels remain consistently low across database versions

**Fig. 3:**
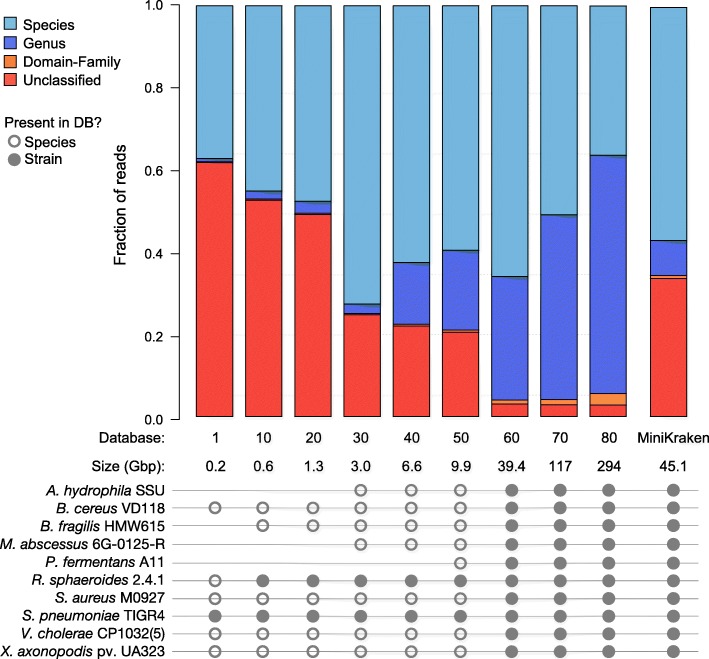
Species-level classifications decreased, and genus-level classifications increased, as bacterial RefSeq grew. Fraction of simulated reads classified at different taxonomic levels, regardless of accuracy, using Kraken against ten databases. The circles below indicate when each genome’s species/strain is in a database. Although the MiniKraken database contains all 10 genomes, it yields results comparable to bacterial RefSeq version 40

Kraken’s own simulated validation set of ten known genomes was searched against nine versions of bacterial RefSeq (1, 10, 20, 30, 40, 50, 60, 70, 80) and the MiniKraken database (4GB version) (Fig. 2). The accuracy of each Kraken run depends on the RefSeq version used in the search (Fig. 2; Table 1). Correct genus-level classifications increased as RefSeq grew, but correct species-level classifications peaked at version 30 and tended to decline thereafter (Fig. 2). The decrease in correct species classifications is due to more closely related genomes appearing over time in RefSeq, making it difficult for the classifier to distinguish them and forcing a move up to the genus level, as that is the lowest common ancestor (LCA). Overall, misclassified species-level calls were consistently rare, as reads were misclassified at the species level an average of 7% of the time (Table 1; Fig. 2). The fraction of reads classified at any taxonomic level, regardless of accuracy, increased as RefSeq grew over time (Fig. 3). However, the fraction of species-level assignments (again, regardless of accuracy) peaked at RefSeq version 30 and began to decline thereafter, while the fraction of genus-level classifications began to increase.

**Table 1:** Fractions of unclassified (FNR or false-negative rate), percent correctly classified (TPR or true-positive rate), and percent misclassified (FPR or false-positive rate.) simulated reads from ten genomes using Kraken against different versions of bacterial RefSeq

			Genus		Species	
Release	Date	FNR	TPR	FPR	TPR	FPR
1	2003-06-30	0.62	0.38	0.00	0.29	0.08
10	2005-03-06	0.53	0.46	0.01	0.38	0.07
20	2006-11-05	0.49	0.49	0.01	0.40	0.08
30	2008-07-07	0.25	0.74	0.00	0.60	0.07
40	2010-05-07	0.22	0.77	0.00	0.54	0.08
50	2011-11-08	0.21	0.78	0.01	0.52	0.07
60	2013-07-19	0.03	0.96	0.00	0.57	0.09
70	2016-03-03	0.03	0.95	0.01	0.42	0.09
80	2017-01-09	0.03	0.94	0.01	0.28	0.08

Bracken was used to re-estimate the abundances of classifications made by Kraken when searching the simulated reads against eight bacterial RefSeq database versions (1, 10, 20, 30, 40, 50, 60, 70). Bracken first derives probabilities that describe how much sequence from each genome is identical to other genomes in the database. This step requires searching a Kraken database against itself with Kraken, which could not be performed for the MiniKraken DB (as there is no FASTA file for this database) or bacterial RefSeq version 80 (as it would require extensive computation for a database that size). Bracken was able to re-estimate species abundances for 95% of the input data using RefSeq version 70, while Kraken only classified 51% of reads at the species level. Because Bracken may probabilistically distribute a single read’s classification across multiple taxonomy nodes, its performance must be measured in terms of the predicted abundances. Bracken typically included the correct species in its re-estimation, but sometimes included incorrect species in the abundance estimation (on average, 15% of reads were associated with a genome outside of the ten knowns).

### Taxonomic classification of difficult to classify genomes over time

**Fig. 4:**
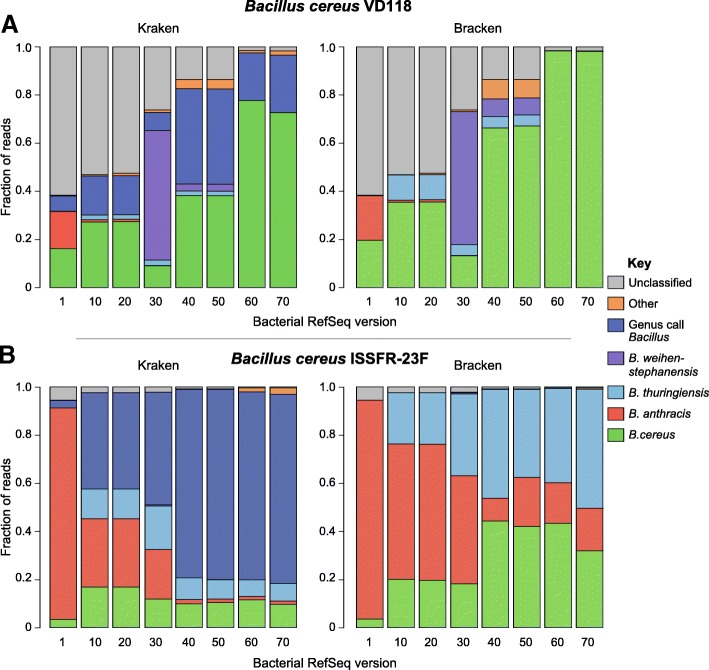
The fraction of reads classified among *Bacillus* species varied depending on which RefSeq version was used. **a** Classifying *B*. *cereus* VD118 reads with Kraken (left) and Bracken (right) against different versions of RefSeq. Species-level classifications varied, and the fraction of unclassified reads decreased with Kraken, as the database grew. Once *B*. *cereus* VD118 appeared in the database (ver. 60), Bracken correctly classified every read. **b** Species-level classifications decrease with Kraken as RefSeq grows using real reads from an environmental *Bacillus cereus* not in RefSeq. Fraction of *B*. *cereus* ISSFR-23F reads classified using Kraken ver. 1.0 (left) and Bracken ver. 1.0.0 (right) against different versions of bacterial RefSeq. Bracken classification pushed all reads to a species-level call, though these classifications were often for other *Bacillus* species

The challenging nature of classifying sequences belonging to the *Bacillus cereus* sensu lato group has been previously documented [14, 15]. The *B*. *anthracis* species within this group is a well-defined monophyletic subclade of the larger *B*. *cereus* group, and the base of the *B*. *anthracis* clade is commonly denoted by a single nonsense mutation in the *plcR* gene [16] which is conserved in all known *B*. *anthracis* genomes and has been shown to confer a regulatory mutation essential for maintaining the pXO1 and pXO2 plasmids that carry the virulence factors characteristic of anthrax [17]. However, not all *B*. *anthracis* strains cause disease in humans, such as *B*. *anthracis* Sterne (missing the pXO2 plasmid), and some *B*. *cereus* strains do cause anthrax-like disease [18], complicating a precise species definition. Thus, it is not surprising that accurate species-level classification within this group has proven challenging for *k*-mer-based methods, especially methods not based on phylogenetic evidence. To demonstrate how difficult sequences from this group have been to classify over time, simulated reads were created for two *Bacillus cereus* strains. The first, *B*. *cereus* VD118, is a strain available in RefSeq version 60 and beyond, and the second, *B*. *cereus* ISSFR-23F [19], was recently isolated from the International Space Station and is not present in any of the RefSeq releases tested. While sharing a relatively high average nucleotide identity (ANI) with *B*. *anthracis* (98.5%), it phylogenetically places outside of the *B*. *anthracis* clade and lacks both the pXO1 and pXO2 plasmids, in addition to other biologically relevant features. Once again, we observed that as bacterial RefSeq grows over time, the number of genus-level classifications made by Kraken increases (Fig. 4). While the number of genus-level calls made by Kraken increases over time, the number of unclassified and misclassified species calls decreases (most commonly *B*. *anthracis*, *B*. *thuringensis*, and *B*. *weihenstephanensis*).

Bracken made species-level predictions for all reads no matter which version of bacterial RefSeq was used (Fig. 4). However, the increased rate of species-level predictions came at the cost of accuracy, as Bracken correctly identified *B*. *cereus* VD118 and *B*. *cereus* ISSFR-23F an average of 72% and 29% of the time, respectively, across RefSeq versions 1 through 70. The fraction of reads assigned to each *Bacillus* species varied substantially from each database tested.

### Taxonomic classification over time with real metagenomes

While simulated metagenomes offer the ability to measure the accuracy of sequence classification, they lack the ability to generate the degree of diversity present in real metagenomic sequences. To understand the trends of taxonomic classification of sequences from real metagenomes, we used Kraken to classify four metagenomes against nine versions of bacterial RefSeq (1, 10, 20, 30, 40, 50, 60, 70, 80).

**Fig. 5:**
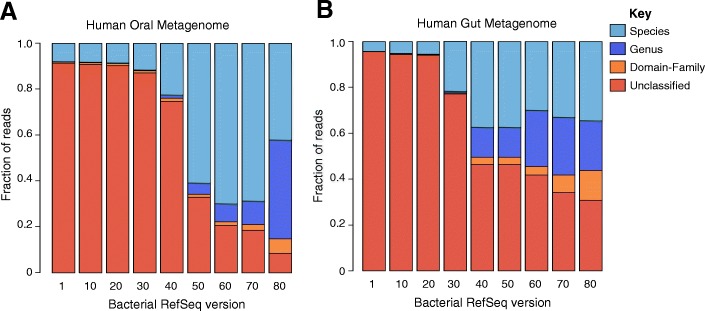
Species-level resolution increased and then tended to decrease in human-associated metagenomes amidst RefSeq growth. Fraction of metagenomic reads classified at different taxonomic levels, regardless of accuracy, using Kraken against nine bacterial RefSeq databases. The human oral metagenome (**a**) exhibited patterns seen in the simulated metagenome (Fig. 3): an increase in species-level classifications, followed by an increase in genus-level classifications. The human gut metagenome (**b**) exhibited a different trend, as species- and genus-level classifications fluctuated and classifications at the family level, and beyond, increased

The two metagenomes constructed from human fecal and oral microbiome samples (Fig. 5a, b) exhibited trends similar to those seen in the simulated datasets: a decrease in unclassified reads and an increase in species-level classifications, followed by a decrease. Additionally, two environmental metagenomes, one from soil and one from oceanic surface water, showed small and steady decreases in the number of unclassified sequences. While only a fraction of the sequences from the soil metagenome were classified (12%), less than half of them were species classifications, whereas the aquatic metagenome produced small, but consistent, increases in the fraction of species classifications.

### CPU/memory performance over time

Historical bacterial RefSeq versions were recreated and used to build Kraken databases with default settings. While most databases were constructed with ease and in less than a day, version 70 required 500 GB of RAM and 2 days (using 64 cores from a single machine containing four E7-8860v4 CPUs and three terabytes of memory), while version 80 required ca. 2.5 TB of RAM and ca. 11 days (using 64 cores from the same machine). Given this trend, future releases will likely require over 4 TB of RAM and weeks of computation to build, putting into question the feasibility of building and profiling *k*-mer databases on future RefSeq versions. Recent studies [20] have suggested alternative approaches for database construction that would help to circumvent future computational bottlenecks.

## Discussion

The results of our study support three conclusions: (i) the diversity of the bacterial RefSeq database is dynamic, and we are in the midst of an unprecedented period of novel species expansion; (ii) the database composition strongly influenced the performance of a taxonomic classification method that relied on LCA; and (iii) the Bayesian method, Bracken, helped mitigate some of the effects, but struggled with novel genomes that had close relatives in the database.

### Database influences on *k*-mer-based taxonomic classification

Using Bracken, the majority of *Bacillus cereus* ISSFR-23F-simulated reads were not correctly assigned to *B*. *cereus* but were more frequently misassigned as *Bacillus anthracis* or *Bacillus thuringiensis* (Fig. 4b). This, in part, is not surprising as two of the three species in this group, *B*. *cereus* and *B*. *thuringiensis*, have no clear phylogenetically defined boundary, though *B*. *anthracis* is phylogenetically distinct from other genomes within this group (*B*. *cereus*, *B*. *thuringiensis)*. Furthermore, any two genomes within the *Bacillus cereus* sensu lato group are likely to be over 98% identical [10]. Given that *k*-mer-based methods are not phylogenetically grounded, but rather based on sequence composition, they are susceptible to misidentification in clades where the taxonomy is in partial conflict with phylogeny, such as the *Bacillus cereus* sensu lato group. One clear example of misidentification within this group was the false identification of anthrax in public transit systems [21, 22]. Improved methods continue to be developed to mitigate these false positives, such as Kraken HLL [23], which reduces false positives by assessing the coverage of unique *k*-mers found in each species in the dataset.

Another observation worth highlighting is that the fraction of simulated reads classified as one of the three *B*. *cereus* sensu lato species varied across database versions (Fig. 4), with the exception of *B*. *cereus* VD118, which was present in RefSeq releases 60 and 70 (Fig. 4a). The variation in species classifications across database versions indicates that even when using the same tools to analyze the same dataset, the conclusions derived from this analysis can vary substantially depending on which version of a database you are searching against, especially for genomes belonging to difficult to classify species (i.e., require phylogenetic-based approaches).

### Imperfect data

The genomic data deluge has helped to expand public repositories with a broader and deeper view of the tree of life but has also brought with it contamination and misclassification [24]. Numerous cases of contamination in public databases are well-documented [25], and databases that continue to harbor these contaminants represent an additional confounding factor for *k*-mer-based methods. While several custom tools have been built to deal with imperfect data [26], there is a need for database “cleaning” tools that can preprocess a database and evaluate it for both contamination (genome assemblies that contain a mixture of species) and misclassified species and strains (genomes that are assigned a taxonomic ID that is inconsistent with its similarity to other genomes in the database). The misclassification issue often is in the eye of the beholder; species have been named based on morphology, ecological niche, toxin presence/absence, isolation location, 16S phylogenetic placement, and average nucleotide identity across the genome. This, coupled with an often ambiguous species concept in microbial genomes due to horizontal gene transfer and mobile elements [27, 28], brings into question the reliance on the current taxonomic structure for assigning names to microbes sequenced and identified in metagenomic samples. To avoid errors due to inconsistencies in the database, classification databases could derive their own hierarchical structure directly from the genomic data, based off of a consistent measurement such as marker gene similarity or average nucleotide identity, rather than taxonomy, and then map back the internally derived hierarchy to widely used taxonomic names [29, 30].

### Generalizability of our findings

We studied the effects of database growth on both simulated and real metagenomic datasets using Kraken, a *k*-mer-based sequence classification method. We also investigated whether Bayesian re-estimation of Kraken results using Bracken helped to mitigate the consequences of this recent “species surge” and allow for species-level assignment. While we only tested one *k*-mer-based classification tool, it is clear that LCA-based assignment (independent of *k*-mers) plays a central role in the increased number of genus-level classifications using recent versions of the RefSeq database. There exist several other tools that apply LCA-based approaches on other databases used for metagenome classification and profiling, such as 16S-based or signature-based tools. An interesting avenue of future work will be to investigate how generalizable these observations are by testing these effects on other databases (e.g., SEED [31], UniProt [32]) and classification approaches (e.g., MetaPhlan [29], MEGAN [8]). Furthermore, as sequencing technologies change, the increased prevalence of long read (e.g., PacBio and Nanopore) and other emerging technologies (e.g., Hi-C [33], 10x [34]) may present new opportunities and challenges to the taxonomic classification of unknown DNA sequences.

## Conclusion

Our findings demonstrate that changes in RefSeq over time have influenced the accuracy of two widely used taxonomic classification and profiling methods. Despite recent progress in *k*-mer-based methods for metagenome profiling and classification, the majority of these tools apply LCA taxonomic assignment and, as a result, are sensitive to changes in strain-to-species and species-to-genera ratios. Bayesian re-estimation approaches are helpful for species- or strain-level prediction but can result in false positives in the presence of unknown species and are computationally prohibitive with larger databases. To reduce the number of unknowns, which can confound existing tools, greater effort should be made to increase the taxonomic breadth of sequenced microbes to better represent the full spectrum of microbial diversity. Lastly, alternative approaches to traditional *k*-mer-based LCA identification methods, such as those featured within KrakenHLL [23], Kallisto [35], and DUDes [36], will be required to maximize the benefit of longer reads coupled with ever-increasing reference sequence databases and improve sequence classification accuracy.

## Methods

### Acquisition of bacterial RefSeq database versions 1 through 80

FASTA files of previous versions of bacterial RefSeq are not publically available for download. Therefore, sequences from previous versions of bacterial RefSeq were acquired using custom scripts (https://github.com/dnasko/refseq_rollback). Briefly, the process involved downloading the current bacterial RefSeq release (ver. 84 as of the date of the beginning of the analysis) FASTA files (ftp.ncbi.nlm.nih.gov/refseq/release/bacteria) and concatenating them into one file. Then, the catalog file associated with the desired version is downloaded (ftp.ncbi.nlm.nih.gov/refseq/release/release-catalog/archive), which contains the identifiers for sequences present in that version of bacterial RefSeq. Sequence identifiers in that version’s catalog file are pulled from the current RefSeq FASTA file and written to a new file. Using the refseq_rollback.pl script, any version of bacterial RefSeq can be created. For this study, only versions 1, 10, 20, 30, 40, 50, 60, 70, and 80 were recreated.

### Taxonomic classification of simulated datasets

Two simulated read datasets were used to test Kraken and Bracken performance with different versions of the bacterial RefSeq database. The first simulated dataset was downloaded from the Kraken website (ccb.jhu.edu/software/kraken) and was previously used in the Kraken manuscript as a validation set [3]. Briefly, this simulated dataset was composed of 10 known bacterial species: *Aeromonas hydrophila* SSU, *Bacillus cereus* VD118, *Bacteroides fragilis* HMW 615, *Mycobacterium abscessus* 6G-0125-R, *Pelosinus fermentans* A11, *Rhodobacter sphaeroides* 2.4.1, *Staphylococcus aureus* M0927, *Streptococcus pneumoniae* TIGR4, *Vibrio cholerae* CP1032(5), and *Xanthomonas axonopodis* pv. Manihotis UA323. Each genome had 1000 single-end reads (101 bp in size) for a total of 10,000 reads. We selected this dataset as it has been widely used as a benchmark for other *k*-mer-based classification methods [3, 7] and represents a breadth of species. This simulated read dataset was classified against each of the recreated bacterial RefSeq databases using Kraken ver. 1.0 with default settings.

Two *Bacillus cereus* genomes were used to test the ability to classify reads from genomes not in the bacterial RefSeq database. The first, *B*. *cereus* VD118, is not present in RefSeq until version 60 and beyond, and the second, a novel *B*. *cereus* genome, *B*. *cereus* ISSFR-23F [19], is never present in any of the RefSeq versions tested. Simulated reads for *B*. *cereus* VD118 were pulled from the 10-organism simulated dataset, while real reads from the sequencing of *B*. *cereus* ISSFR-23F were downloaded from the SRA (SRR3954740) and 10,000 random reads were selected using a script (“pick_random_reads.pl” in github.com/dnasko/refseq_rollback/). We decided to use these genomes as they are members of the *B*. *cereus* sensu lato group, containing a collection of species that are known to be challenging for *k*-mer methods to distinguish between [21, 22]. These datasets were classified with Kraken (ver. 1.0) and Bracken (ver. 1.0.0) [10] both with default settings (Bracken “read-length” set to 101).

### Taxonomic classification of real metagenomic datasets

To assess classification trends in real metagenomic data, two shotgun metagenomes were used: a fecal metagenome (SRS105153) and oral metagenome (SRS050029) from the Human Microbiome Project [37]. Additionally, a soil (SRR5381886) [38] and aquatic (ERR315857) [39] metagenome were analyzed to provide some environmental insights. Reads from these metagenomes were downloaded from the NCBI sequence read archive (SRA) and quality filtered using Trimmomatic ver. 0.23 (leading:20, trailing:20, slidingwindow:4:30 minlen:40) [40]. Filtered reads from only the left pair were then classified using Kraken ver. 1.0 with default settings.

### Running Bracken on Kraken output

Bracken (ver. 1.0.0) was run on the output of each Kraken search (except for release 80 and KrakenMiniDB). Default parameters were used except for “read-length,” which was set to 101.

### Bacterial RefSeq diversity metric calculations

Diversity metrics were calculated for every version of bacterial RefSeq (1–89) by parsing the catalog files for each version. The “dump_taxonomy_species.pl” script in the refseq_rollback repository was used to convert the NCBI taxonomy ID on each line to its species name. Using this file, an operational taxonomic unit (OTU) table was constructed at the species- and genus-levels using the “create_otu_table.pl” in the refseq_rollback repository. The OTU table was imported to QIIME1 (ver. MacQIIME 1.9.1-20150604) [41]. Diversity metrics (Simpson, Shannon, Richness) were calculated using the “alpha_diversity.py” script and plotted using the R base package. Counts and diversity metrics from RefSeq versions 57, 58, and 59 were excluded from the analysis, as these versions proved to be outliers. This was due to a reorganization of the bacterial RefSeq collection in these versions (for further reading, see the section “CPU/memory performance over time” in the release notes for these versions, e.g., “RefSeq-release57.txt”).

## Abbreviations

LCA: Lowest common ancestor
OTU: Operational taxonomic unit
